# Suppurative Pylephlebitis

**Published:** 1907-03-30

**Authors:** 


### Points in Diagnosis.

### SUPPURATIVE PYLEPHLEBITIS.

By suppurative pylephlebitis is meant that condition of multiple small abscesses in the liver which results from bacterial infection of the branches into which the portal vein divides within the liver. Another name for it is portal pyæmia. It is not a common condition, it is seldom diagnosed correctly. In the great majority of cases, notwithstanding the severity of the illness, the cause of the patient's symptoms and fever is only ascertained at the autopsy. It is on this account that it seems worth while to discuss the subject, notwithstanding its rarity; for it is only by being familiar with it that better diagnosis can be expected. It may be urged that nothing can be done to cure the condition; this is true; but at the same time it is very distressing to have a patient under one's care, very ill, suffering from rigors and continued pyrexia, and yet not to be able to give a name to the disease, nor to be sure of the prognosis.

Suppurative pylephlebitis, or portal pyæmia, is never a primary condition; the micro-organisms are always carried to the liver from some source outside that organ; and any inflammatory lesion of the tracts drained by the portal vein may give rise to the infection. The primary trouble may be in the stomach or duodenum—a leaking ulcer; in the gall-bladder—gall-stones or growth, though these may give rise to a different variety of multiple abscesses in the liver—namely, suppurative cholangitis; in the jejunum and ileum, though these are very seldom indeed the source of the infection, neither tuberculous nor typhoidal ulceration of the bowel being followed by it; in the vermiform appendix—nearly half the cases are due to appendicitis; in the colon—ulcerative colitis; in the rectum—necrosing hæmorrhoids; in the pelvis—secondary to pyosalpinx, abortion, and similar causes of pelvic peritonitis.

In regard to causation, the great point to bear in mind is that appendicitis is at the bottom of nearly half the cases. Moreover, it is almost always after comparatively mild appendicitis, not after those attacks which are severe enough to require appendicectomy or the incision and drainage of an abscess within a comparatively short time of the onset of the trouble. The appendicitis probably leads to thrombosis of the appendicular veins; organisms, usually the bacillus coli communis, grow in the blood-clot, and some of them get carried to the liver by the portal vein. The number of secondary abscesses which result depends upon the number of hepatic branches of the portal vein into which colonies of bacilli get carried. It is a true portal pyæmia, for it hardly ever happens that there is a continuous thrombosis to the portal vein from the site of the original septic thrombosis. Another remarkable thing is that the liver nearly always acts as a most efficient filter to the bacilli, intercepting them and preventing them from getting into the hepatic veins and the general circulation. Why it should be the milder cases of appendicitis, and not the more severe, that are most liable to give rise to portal pyæmia is quite unknown, though the pathological fact is of considerable clinical import.

The symptoms as a rule are vague, the one constant factor being continued pyrexia, with occasional rigors. There is nothing definite about the temperature chart beyond the fact that there is pyrexia; this may be continuous, irregular, intermittent, or remittent; it may not exceed 102° F. or it may rise as high as 107° F. The patient usually perspires freely at the time of the rigors; and though the appetite may for some time remain fair, almost all the patients gradually waste. The duration is comparatively long—that is to say, longer than one might expect; in some cases it has lasted for as much as 296 days; in a few cases it has been no longer than a week, but the average duration is eight weeks.

The difficulty is to know what is the cause of the pyrexia and rigors in the particular case that one is attending. The points to be on the look out for are: (1) Tenderness in the hepatic region; (2) moderate and uniform enlargement of the liver; (3) rapid action of the heart and rapid rate of respiration; (4) jaundice. If all these, including jaundice, are present, the possibility of suppurative pylephlebitis should occur to one, the main difficulties being to diagnose it from gall-stones, carcinoma of the gall-bladder or liver, cirrhosis of the liver, and subacute pancreatitis. As a rule there is no real colic in cases of portal pyæmia, so that gall-stones and pancreatitis may in this way be excluded; the absence of any mass or lump will be an argument against new growth; and cirrhosis can hardly be considered when there is no history of alcoholism or hæmatemesis.

It is, however, the exception rather than the rule to get jaundice with suppurative pylephlebitis; were jaundice always present the diagnosis would be a great deal easier. Tenderness over the liver and moderate enlargement of the organ are much more constant signs; they are not, however, much more marked than they are in many cases of nutmeg liver from heart-failure. It is very easy, therefore, to attribute both the pyrexia and rigors and the enlargement and the tenderness of the liver in these cases to cardiac trouble, and to make a diagnosis of fungating endocarditis.

In other cases the tenderness may not be restricted to the hepatic region, but may be spread more widely over the abdomen; in such cases typhoid fever may be suspected. This should be readily excluded on three accounts, namely: (1) The blood serum will give no positive Widal's reaction; (2) there will almost certainly be rigors, which are very rare in typhoid fever; (3) the pulse-rate will be high in proportion to the temperature, whereas in typhoid fever the pulse-rate, though rapid, is usually slow in proportion to the temperature, at least during the first two weeks; for example, with a temperature of 104° F. the physiological pulse-rate should be 125 per minute; with typhoid fever it is very often not much over 100, and may be less; whereas with suppurative pylephlebitis it will often be 130 to 140.

In some of the cases the pain and tenderness are not referred to the abdomen at all, but to the right side of the chest. There may be no abnormal physical signs to be detected at the base of the right lung, and yet it will be difficult to exclude diaphragmatic pleurisy, deep-seated pneumonia, empyema, or sub-diaphragmatic or hepatic abscess. In some cases the pleura and lung do actually become infected, and an empyema may be found and operated upon with little or no improvement in the patient's condition. Sub-diaphragmatic abscess will usually cause the typical dome-shaped dullness at the base of the right lung, and since the causes of suppurative pylephlebitis are many of them also causes of sub-diaphragmatic abscess, the difficulty in deciding which of the two is present is extremely great. This, however, is no great matter, for if the differential diagnosis has been narrowed down to this point, it will nearly always be correct to operate to find out; because operation cannot make the prognosis of portal pyæmia worse, and will perhaps cure any large local abscess. It is in the cases where there are no localising symptoms and signs that the medical man will find it hardest to give either a diagnosis or prognosis, and will be in the gravest doubt as to what line of treatment to adopt.

In most abdominal conditions the respiration rate is not increased out of proportion to the temperature. This is otherwise in suppurative pylephlebitis; the respiration rate may be 40 or over when the temperature is 104° F. or less; and on this account pneumonia may be suspected. There may be no abnormal physical signs in the lungs, but this, of course, does not exclude pneumonia; one sometimes sees cases of lobar pneumonia, typical in all other respects, run their course without any signs of consolidation reaching the surface. The main point of distinction between portal pyæmia and lobar pneumonia is the long persistence of the illness; slighter aids in diagnosis are (1) the testing of the urine for chlorides, these being much more diminished in lobar pneumonia than in any other condition; and (2) examination of the sputum, which would not be the typical rusty sputum of pneumonia if the condition were portal pyæmia, and would not contain large numbers of pneumococci.

It might be thought that a bacteriological examination of the blood taken from a vein might be of service in making a diagnosis. In a few cases this may be so; but, as has been mentioned, the liver in most cases acts as a most efficient filter, preventing the micro-organisms from getting past it into the general circulation. The liver itself may be crowded with portal abscesses, and yet there may be no abscesses anywhere else in the body.

The above has necessarily been but a brief account of the condition known as suppurative pylephlebitis; the diagnosis is difficult, but not impossible; one of the chief reasons for not making the diagnosis is that the possibility of the condition is apt not to pass through one's mind even when the case is in front of one. Pylephlebitis should always be remembered as a possible explanation in any obscure condition associated with prolonged pyrexia and rigors, particularly if the liver is tender and uniformly enlarged; and seeing that the condition is never primary, but always preceded by some inflammatory lesion of one of the viscera from which the blood drains into the portal vein, it is extremely important to obtain a very accurate history of how the illness began. Comparatively mild appendicitis is the most common precursor of the condition; in all suspicious cases it will be a great assistance to the diagnosis of portal pyæmia if the patient gives a history of an attack of apparently unimportant pain in the right iliac fossa during the week or ten days preceding the onset of the severer and more obscure symptoms.

